# Sasanquasaponin inhibited epithelial to mesenchymal transition in prostate cancer by regulating the PI3K/Akt/mTOR and Smad pathways

**DOI:** 10.1080/13880209.2022.2123931

**Published:** 2022-10-07

**Authors:** Wenfeng Li, Yuanshen Mao, Bao Hua, Xin Gu, Chao Lu, Bin Xu, Weixin Pan

**Affiliations:** a Department of Urology, Shanghai Ninth People’s Hospital, Shanghai Jiao Tong University School of Medicine, Shanghai, China; b Department of Urology, Hainan Western Central Hospital, Danzhou, China

**Keywords:** Traditional Chinese medicine, signalling pathway, adhesion, migration, invasion

## Abstract

**Context:**

Sasanquasaponin (SQS) is a commonly used traditional Chinese medicine proved to have a wide range of pharmacological functions.

**Objective:**

The objective of this study is to explore the effect and underlying mechanism of SQS in the treatment of prostate cancer (PC).

**Materials and methods:**

PC cell lines (22Rv1 and PC-3) were treated with SQS (0, 0.5, 1, 2, and 4 μM) for 12 or 24 h. The viability of cells was evaluated, while the mRNA and protein levels of epithelial to mesenchymal transition (EMT)-related genes in PC cell lines were measured (Groups: Control, TGF-β1, TNF-α, TGF-β1 + TNF-α, and TGF-β1 + TNF-α + SQS). The migration and invasion abilities of PC cell lines were evaluated (Groups: Control, SQS). Finally, the antitumour effect of SQS (25, 50,100, and 200 mg/kg) in BALB/c nude mice (6 weeks, 18–20 g) was evaluated (Groups: Control, Vehicle, 25, 50,100, and 200 mg/kg SQS). The study duration was 1 month.

**Results:**

SQS inhibited the viability and the number of colonies of 22Rv1 or PC-3 cells. The IC_50_ of SQS of 12 and 24 h in these two cells was 3.25, 1.82, 4.76, and 4.70 μM, respectively. SQS inhibited the adhesion, migration, and invasion of PC cells. It also inhibited the expression of EMT-related markers of PC cells. The PI3K/Akt/mTOR and Smad2/3 signalling pathways were activated in the process of EMT, and SQS could significantly reduce the activation of the PI3K/Akt/mTOR and Smad2/3 pathways. Finally, SQS inhibited the growth of xenograft tumours *in vivo*.

**Conclusions:**

SQS inhibited EMT in PC by regulating the PI3K/Akt/mTOR and Smad pathways.

## Introduction

Prostate cancer (PC) is the most frequently diagnosed malignant cancer and the second leading cause of cancer-associated mortality in men worldwide (Siegel et al. 2017). Due to the improvements in therapeutic strategy and the advances in diagnostic technology, the 5-year survival rate of patients with primary PC was >99% (Mukherji et al. 2013; Siegel et al. 2015; Evans 2018). However, high mortality rates and poor outcomes were observed in patients with PC with distant metastasis (Siegel et al. 2015). Therefore, due to the incomplete understanding of the mechanisms controlling the pathogenesis of PC, it is necessary to explore more effective therapeutic agents and novel biomarkers for PC clinical therapy in order to improve survival rates.

As PC is a major disease treated in our department (Department of Urology), we focussed on the exploration of potential medicine for PC and the underlying mechanism, as well as new therapeutic target for it. Sasanquasaponin (SQS), a biologically active ingredient extracted from the defatted seeds of *Camellia oleifera* Abel (Theaceae), is a commonly used traditional Chinese medicine. Previous studies have demonstrated that SQS exhibits a wide range of pharmacological functions, including cardio-protective, anti-inflammatory, and antioxidative functions (Chen et al. 2007; Ye et al. 2013; Qiu et al. 2016). For instance, Chen et al. (2007) found that SQS treatment could protect rat cardiomyocytes against oxidative stress induced by anoxia-reoxygenation injury. Some studies have identified that SQS may also exert anticancer properties in multiple tumours, including breast and liver cancer (Chen et al. 2013; Zeng et al. 2015). Chen et al. (2013) reported that SQS may contribute to the cell cycle arrest and apoptosis of human breast cancer cells *in vitro*. Furthermore, Zeng et al. (2015) also demonstrated that SQS treatment could inhibit the proliferation of liver cancer via activating Bcl-2, Bax, and caspase-3. However, the role and underlying mechanism involved in the effects of SQS on PC have not been fully elucidated to date.

Neoplastic metastasis is a complex process that is regulated by several molecular mechanisms. Epithelial to mesenchymal transition (EMT) has been identified as a key factor associated with the invasion and metastasis of several malignant tumours, including PC (Odero-Marah et al. 2018). During the progression of EMT, the matrix junctions and apical–basal polarity of epithelial cells are disrupted, thus leading to low proliferation, and high migration, invasion, and survival of cancer cells (Lamouille et al. 2014). EMT can be induced via several upstream signalling molecules and pathways, including the PI3K/Akt and Smad signalling pathways. For example, Xu et al. (2021) reported that methyltransferase-like 14 activate PTEN to regulate the EMT of renal tubular cells via the PI3K/Akt signalling pathway. Additionally, double cortin-like kinase 1 promotes EMT via the Smad pathway in colorectal cancer (Liu et al. 2018).

Based on the previous reports, we hypothesized that SQS treatment may also show an inhibitory impact in PC. As the EMT plays an important role in the development of PC, we hypothesized that SQS may also have an impact on EMT in PC cells, and the PI3K/Akt or Smad also play an important role in the EMT progression of PC and is involved in the antitumour effect of SQS. The inhibitory impact of SQS on the viability, adhesion, migration, and invasion of PC cell lines was analysed. The results suggested that the PI3K/Akt/mTOR and Smad signalling pathways may be responsible for inhibiting the EMT of PC cell lines induced by SQS. Furthermore, SQS also inhibited the growth of xenograft tumours *in vivo*. These results indicated that SQS treatment could be a promising therapeutic strategy in PC.

## Materials and methods

### Cell culture

Human prostate carcinoma cell lines (22Rv1 and PC-3) were obtained from the Cell Bank of the Chinese Academy of Sciences. In brief, cells were cultured in RPMI-1640 medium (Hyclone; Cytiva) supplemented with 10% FBS (Gibco; Thermo Fisher Scientific, Inc.) at 37 °C with 5% CO_2_ in a humidified incubator. The medium was changed every 2 days. Cells were used for experiments between passages 2 and 5.

**Figure 1.**
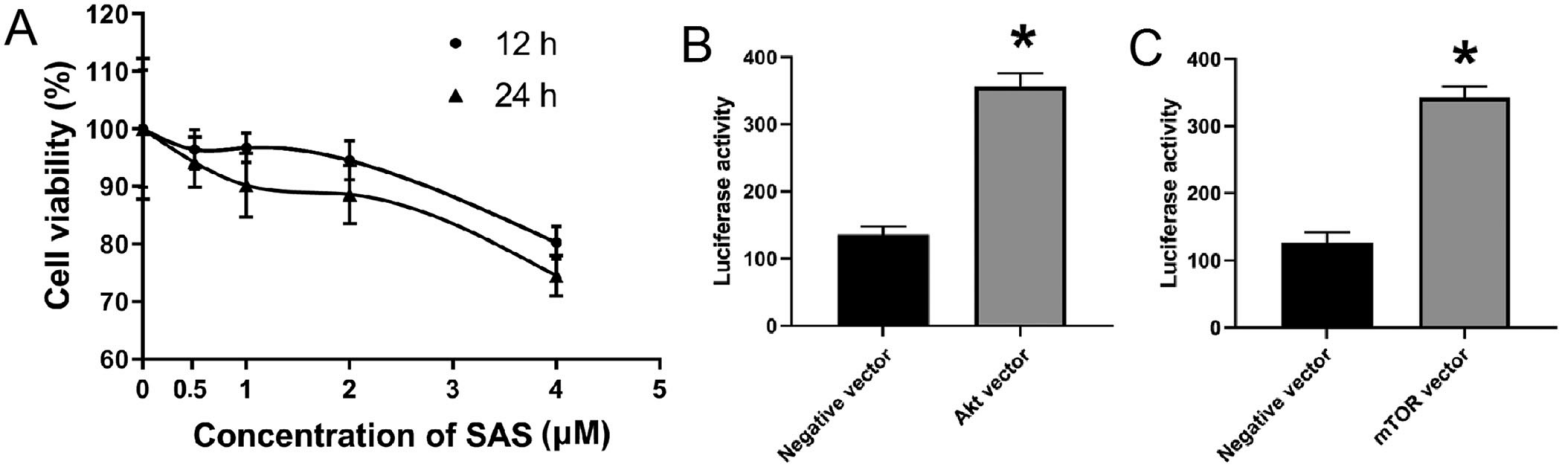
Effect of SQS on the viability of RWPe-1 cells and the result of luciferase activity. The toxicity of SQS extract was tested in RWPe-1 cells. *p < 0.05 compared with negative control. SQS, sasanquasaponin.

22Rv1 and PC-3 cells were treated with TGF-β1 (10 ng/mL, Beyotime; Institute of Biotechnology) and/or TNF-α (10 ng/mL, Beyotime; Institute of Biotechnology) for 24 h at 37 °C. 22Rv1 and PC-3 cells were also stimulated with SQS, which was extracted, isolated, and purified from the defatted seeds of Chinese medicinal plant *C. oleifera* using the method described by Chen et al. (2007). Briefly, the defatted seeds of *C. oleifera* was grounded and treated with boiling water. The extract from the seeds–water mixture was concentrated and treated with ethyl acetate and *n*-butanol, and then fractionated via silica gel column chromatography. Finally, the crude crystals were collected and purified to obtain SQS. SQS was dissolved in dimethyl sulphoxide (DMSO) at 25 mM as a stock solution and was stored at −20 °C. The purity of SQS was examined using reverse-phase high-performance liquid chromatography and was determined to be 97.32%. The results of HPLC and NMR are shown in Supplementary Figure S1. Next, SQS was dissolved in DMSO at 25 mM as a stock solution and was stored at −20 °C. When used, SQS was added to the cell culture medium to obtain specific concentration (0, 0.5, 1, 2, and 4 μM) and the cells were treated by SQS for 12 or 24 h according to the study of Liang et al. (2019). The toxicity of this extract was tested in RWPe-1 cells. After RWPe-1 cells were treated with 0.5, 1.0, and 2.0 μM SQS for 12 or 24 h, the viability was not significantly different compared with control. After RWPe-1 cells were treated with 4.0 μM SQS for 12 or 24 h, the viability was significantly decreased compared with control. The results are shown in Figure 1(A).

### Plasmid transfection

Akt or mTOR cDNA were cloned into the pcDNA.1 (+) expression vector to construct the corresponding plasmids. Next, the pcDNA-Akt, pcDNA-mTOR, and control plasmids were transfected into 22Rv1 cells using Lipofectamine™ 2000 reagent (Invitrogen; Thermo Fisher Scientific, Inc.) according to the manufacturer’s protocol. Briefly, parental 22Rv1 cells (4 × 10^5^ cells/well) were divided into six-well plates and grown until 75% confluent. Next, each well was treated overnight with a premixed solution containing Lipofectamine™ 2000 reagent and plasmid at a 5 μL:2 μg ratio. The luciferase activity was measured at 48 h after the plasmid transefection to verify transfection using a dual-luciferase activity reporter assay system (Promega). Renilla luciferase activity was normalized to firefly luciferase expression in each sample. The results are shown in Figure 1(B,C).

### Cell viability assay

The proliferation of 22Rv1 or PC-3 cells was assessed using a cell counting kit-8 (CCK-8) assay (Abcam) according to the manufacturer’s instructions. Briefly, each group of cell suspensions was divided into 96-well plates (5 × 10^4^ cells/mL) and incubated with RPMI-1640 medium for 24 h. Next, the medium was changed to a mixture of 90 µL fresh medium and 10 µL CCK-8 solution. Subsequently, the 96-well plates were incubated for 2 h at 37 °C, and the optical density value of each well was determined at 450 nm with a microplate reader (BioTek Instruments, Inc.).

### Colony formation assay

22Rv1 or PC-3 cells were digested using a trypsin-EDTA solution (MilliporeSigma; Merck KGaA) and concentrated to a density of 150 cells/mL. Next, a 2 mL cell suspension (300 cells/well) was added into six-well plates, treated with SQS or vehicle (saline) for 24 h, and rinsed twice with PBS. Cells were then fixed with 4% paraformaldehyde and stained with haematoxylin and eosin. The number of colonies containing >50 cells were counted using a light microscope (ZeissGmbH).

### Transwell invasion and migration assay

First, 100 μL of 1:8 DMEM-diluted Matrigel (MilliporeSigma; Merck KGaA) was used to pre-coat the membrane at 37 °C for 6 h before the cells were seeded. 22Rv1 or PC-3 cells (2 × 10^4^ cells) were suspended in 100 μL of serum-free RPMI-1640 medium and added to a Transwell chamber wells (Corning, Inc.). Next, the bottom of the chambers was filled with 500 μL RPMI-1640 medium containing 5% serum (Beyotime, Shanghai, China) to act as a chemoattractant. After 48 h, the non-invaded cells were removed using a cotton swab, while the invaded cells on the bottom of the chambers were fixed with 4% paraformaldehyde. The invaded cells were stained with 0.1% crystal violet, and visualized using a light microscope (Zeiss GmbH).

The transwell migration assay was performed as described above, except that no Matrigel was used. 22Rv1 or PC-3 cells (2 × 10^4^ cells/well) were seeded in the upper chamber in 100 μL of serum-free medium, and 500 μL of medium containing 5% serum (Beyotime, Shanghai, China) was added to the lower chamber. After incubated for 48 h at 37 °C, the cells at the upper surface of the membrane were removed, while the cells on the lower surface of the membrane were stained with 0.1% crystal violet, and visualized.

### Wound healing assay

22Rv1 or PC-3 cells were cultured in T75 plates (Thermo Fisher Scientific, MO, USA) until reaching 90% confluence. Next, the cells were cultured in a 96-well plate (5 × 10^4^ cells/well) in the RPMI-1640 medium with 2% foetal bovine serum. After 24 h, the plate was scratched in the centre using a pipette tip. The shredded cells were removed by washing with cold PBS, and the RPMI-1640 medium was replaced with serum-free medium. The cells migrated into wound surface, which was considered as the process of *in vitro* healing. After 24 h, the visualization of the closure of the wound was performed under a light microscope (Zeiss GmbH). The rate of wound healing = [(the wound width of 0 h − 24 h)/0 h wound width] × 100%.

### Western blotting

RIPA buffer (MilliporeSigma; Merck KGaA) was used to extract total proteins from cultured 22Rv1 or PC-3 cells. Proteins were quantified with a BCA method and separated by 12% SDS-PAGE, and transferred onto PVDF membranes (GEHealthcare). The membranes were blocked in 5% non-fat dry milk diluted with TBST (Tris–HCl 20 mmol/L, NaCl 150 mmol/L, pH 7.5, 0.1% Tween 20) at room temperature for 1 h and washed with TBST for three times. Subsequently, specific primary antibodies (anti-Vimentin, anti-N-cadherin, anti-a-SMA, anti-E-cadherin, anti-Claudin 1, anti-GAPDH, anti-p-Akt, anti-Akt, anti-p-mTOR, anti-mTOR, anti-PTEN, anti-p-Smad2, anti-Smad2, anti-p-Smad3, anti-Smad3, anti-p-Smad2/3, anti-Smad2/3, and anti-laminB, Sigma, USA) were incubated with the membranes at 4 °C overnight. Next, the membranes were washed with TBST for three times and incubated with the secondary antibodies at room temperature for 2 h. Proteins were visualized using chemiluminescence using ECL luminescence reagent (Absin Bioscience Inc., Shanghai, China). The band intensities were quantified using Image-Pro Plus 6.0 (Media Cybernetics, Inc.). GAPDH was used as a loading control for normalization. The primary antibodies and secondary antibodies used in western blotting are included in Table 1.

**Table 1.** Antibodies used for western blot analysis.

Reagent	Source	Identifier
Primary antibodies		
Anti-vimentin antibody, rabbit monoclonal	Abcam	ab92547
Anti-N-cadherin antibody, rabbit monoclonal	Abcam	ab76011
Anti-α-SMA antibody, rabbit polyclonal	Affinity	AF1032
Anti-E-cadherin antibody, rabbit monoclonal	Abcam	ab40772
Anti-Claudin antibody, rabbit monoclonal	Abcam	ab211737
Anti-phospho-EGFR antibody, rabbit monoclonal	Cell Signaling Technology	3777
Anti-AKT antibody, rabbit monoclonal	Cell Signaling Technology	4691
Anti-phospho-AKT antibody, rabbit monoclonal	Cell Signaling Technology	4060
Anti-mTOR antibody, rabbit polyclonal	Invitrogen	PA5-34663
Anti-phospho-mTOR antibody, rabbit monoclonal	Cell Signaling Technology	5536
Anti-Smad2 antibody, rabbit monoclonal	Abcam	ab280888
Anti-phospho-Smad2 antibody, rabbit monoclonal	Cell Signaling Technology	3108
Anti-Smad3 antibody, rabbit monoclonal	Abcam	ab40854
Anti-phospho-Smad3 antibody, rabbit monoclonal	Cell Signaling Technology	9520
Anti-TBP antibody, rabbit monoclonal	Abcam	Ab171969
Anti-PTEN antibody, rabbit monoclonal	Abcam	ab267784
Anti-GAPDH antibody, rabbit polyclonal	Abcam	ab9485
Secondary antibodies		
Goat Anti-Rabbit IgG H&L (HRP)	Abcam	ab97051

### qPCR procedure

Total RNA from 22Rv1 or PC-3 cells was extracted with a DNA/RNA/Protein Extract Kit (Thermo Fisher Scientific, Inc.), and cDNA was synthesized with a Reverse Transcription Kit (Shanghai Yeasen Biotechnology Co., Ltd.) on a C1000 Touch Thermal Cycler (Bio-Rad Laboratories, Inc.) following the manufacturer’s instructions. qPCR was conducted on a ViiA 7 Real-Time PCR System (Thermo Fisher Scientific, Inc.). The thermocycling conditions used in PCR were accordance with the manufacturer’s instructions. The relative expression levels of target genes were calculated using the 2^−ΔΔCq^ method, and GAPDH was used as a control for normalization. The primers used for qPCR are listed in Table 2.

**Table 2.** Primers used for PCR.

Gene	Forward	Reverse
E-cadherin	CAGAACAGAAATACATCTCAGGGC	TCGTGCACCGAAAGTTTCAA
Claudin 1	TTGGGCTTCATTCTCGCCTT	CTGGCATTGACTGGGGTCAT
Vimentin	CCGCACATTCGAGCAAAGAC	AAGCGCACCTTGTCGATGTA
N-cadherin	GGGAAATGGAAACTTGATGGCA	GGAGGGATGACCCAGTCTCT
α-SMA	AAAGCAAGTCCTCCAGCGTT	TTAGTCCCGGGGATAGGCAA
GAPDH	CTACAATGAGCTGCGTGTGGC	CAGGTCCAGACGCAGGATGGC

### Animal experiments

BALB/c nude mice (*n* = 30; male and female; age, 6 weeks; body weight, 18–20 g) were obtained from Shanghai Model Organisms Center, Inc. Mice were housed in an environment with a temperature of 22 ± 1 °C, a relative humidity of 50 ± 1% and a 12 h light/dark cycle. Mice were allowed access to standard rat food and water *ad libitum*. The whole experimental protocol followed the ‘Guide for the Care and Use of Laboratory Animals, 8th Edition’ and was approved by the Institutional Animal Care and Use Committee of Shanghai Jiao Tong University School of Medicine (No. 201956.5892).

For xenograft tumour model, 1 × 10^7^ 22Rv1 cells were inoculated subcutaneously into nude mice. Then, mice were subjected to intragastric administration of normal saline vehicle (0.2 mL) and daily exposure to SQS for 6 weeks at specific doses after anaesthesia with 6% (induction) and 2% (maintenance) isoflurane. The control group was treated with vehicle alone. Individual body weights were recorded every other day. After 15 days, the mice were anaesthetized and sacrificed by cervical dislocation, and the tumours were separated and weighed. The maximum tumour volume observed in the study was 2.0 cm^3^.

For Ki-67 staining, tumours were fixed in 4% paraformaldehyde, dehydrated, embedded in paraffin, sectioned and dried overnight at 37 °C. The sections were then deparaffinized in xylol, rehydrated in alcohols and rinsed in distilled water. Next, the sections were treated by heat-induced epitope retrieval. Endogenous peroxidase activity was blocked by 2% H_2_O_2_. The slides were then incubated with monoclonal anti-Ki-67 antibody (eBioscience™, Invitrogen, USA) for 2 h. The sections were rinsed and incubated for 30 min with a biotinylated anti-mouse IgG secondary antibody (Invitrogen, USA), then with streptavidin–peroxidase conjugate. Brown staining located in nucleus was evaluated as Ki-67-positive staining. Ki-67 labelling percentage was calculated as number of Ki-67 labelled nuclei/total number of cells counted × 100%.

### Statistical analysis

Each experiment was performed three times, and measured data were presented as the mean ± SD. Statistical analysis was performed with SPSS 21.0 statistical software (IBM Corp.). The data were analysed using a paired Student’s *t*-test or one-way ANOVA followed by a Dunnett’s *post hoc* test. *p* < 0.05 was considered to indicate a statistically significant difference.

## Results

### SQS inhibited the viability of PC cell lines

**Figure 2.**
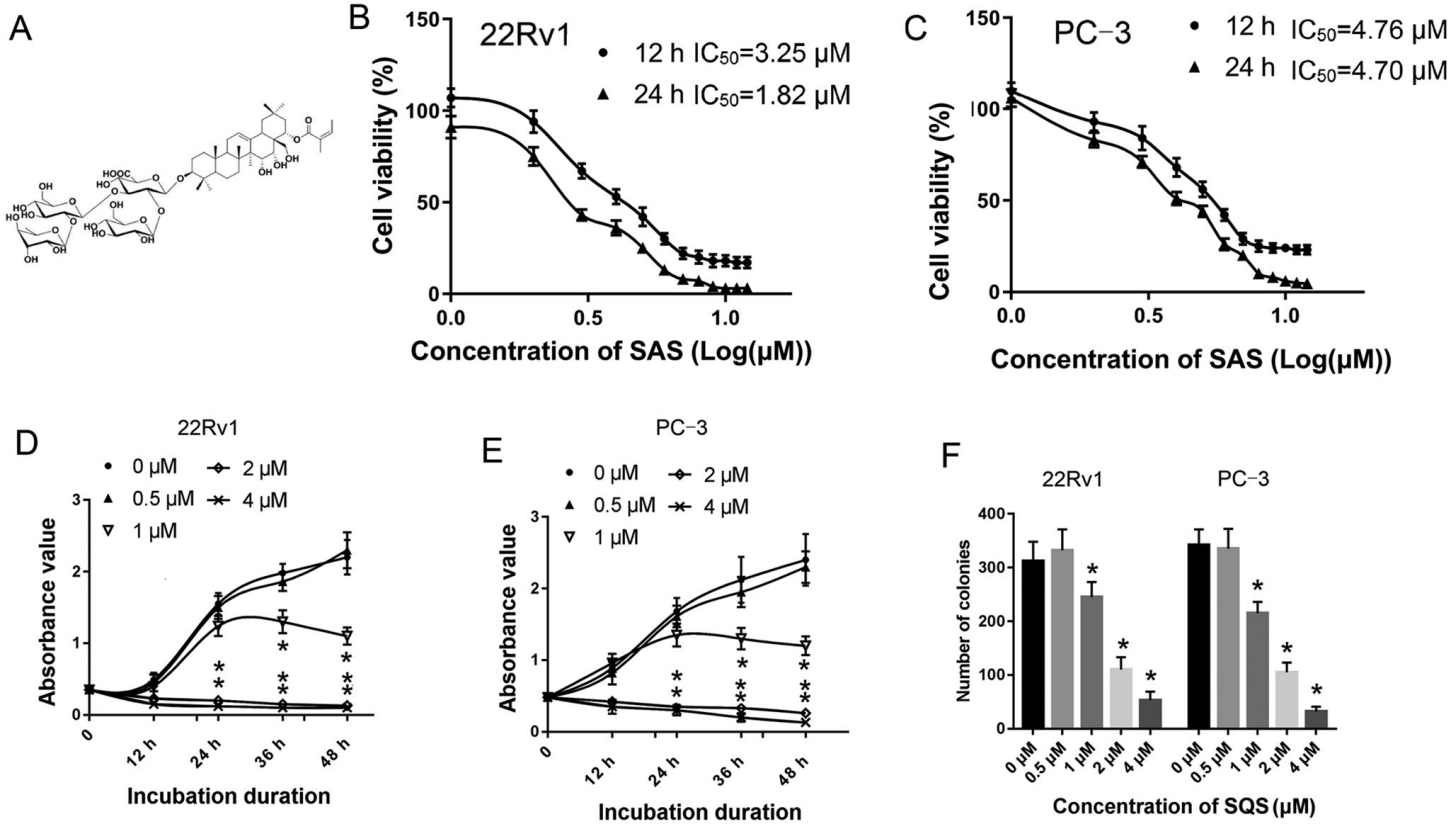
SQS inhibited the viability of prostate cancer cell lines. (A) Chemical structure of SQS. The purity of SQS was examined using reverse-phase high-performance liquid chromatography. SQS was dissolved in dimethyl sulphoxide (DMSO) at 25 mM as a stock solution and was stored at −20 °C. When used, SQS was added to the cell culture medium to obtain specific concentration. Viability of (B) 22Rv1 and (C) PC-3 cells after being treated with different concentrations of SQS. Viability of (D) 22Rv1 and (E) PC-3 cells after being incubated with SQS for different periods of times. (F) Number of colonies formed in 22Rv1 or PC-3 cells after treatment with different concentrations of SQS. Each experiment was performed three times, and the data are presented as the mean ± SD. *p < 0.05 vs. 0 μM group. SQS, sasanquasaponin. Next, SQS was dissolved in dimethyl sulphoxide (DMSO) at 25 mM as a stock solution and was stored at −20 °C. When used, SQS was added to the cell culture medium to obtain specific concentration (0, 0.5, 1, 2, 4 μM) for 12 or 24 h.

The chemical structure of SQS is shown in Figure 2(A). To elucidate the inhibitory effects of SQS on PC, different concentrations of SQS were used to stimulate PC cell lines (22Rv1 or PC-3) for 12 or 24 h. It was found that the viability of the PC cell lines showed an inhibition after treatment with SQS for 12 or 24 h (Figure 2(B,C)). The IC_50_ of SQS of 12 and 24 h in 22Rv1 cells was 3.25 and 1.82 μM, respectively; The IC_50_ of SQS of 12 and 24 h in PC-3 cells was 4.76 and 4.70 μM, respectively. The inhibitory effects of SQS on PC cell lines after incubation for different periods of times were evaluated. As shown in Figure 2(D,E), the viability of 22Rv1 or PC-3 cells could also be suppressed by SQS.

Additionally, a colony formation assay was used to evaluate the negative impact of SQS on PC cell lines. After treatment with SQS, the number of colonies in the PC cell lines decreased (Figure 2(F)). These results suggested that SQS could inhibit the viability of PC cells *in vitro*.

### SQS inhibited the adhesion, migration, and invasion of PC cell lines

**Figure 3.**
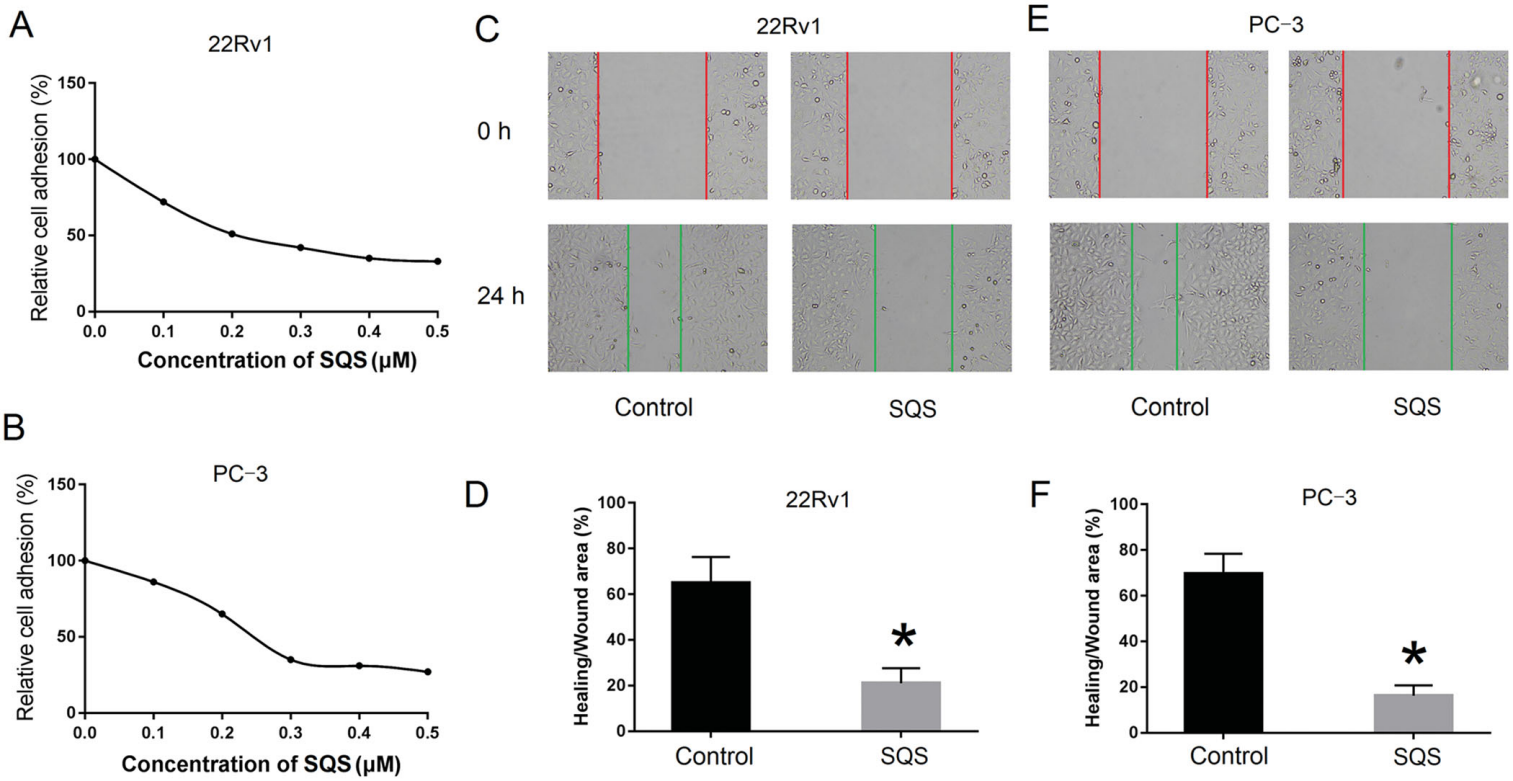
SQS inhibited the adhesion and migration abilities of prostate cancer cell lines. (A and B) Adhesion ability of (A) 22Rv1 and (B) PC-3 cells after treatment with different concentrations of SQS. (C) Migratory ability of 22Rv1 cells after treatment with different concentrations of SQS. (D) Ratio of healing to wounded areas in 22Rv1 cells after treatment with different concentrations of SQS. (E) Migratory ability of PC-3 cells after treatment with different concentrations of SQS. (F) Ratio of healing to wounded areas in PC-3 cells after treatment with different concentrations of SQS. Each experiment was performed three times, and the data are presented as the mean ± SD. *p < 0.05 vs. control. SQS, sasanquasaponin.

The characteristics of tumour metastasis of PC cell lines after treatment with SQS were evaluated. Cells were treated with 0.5 µM SQS for 24 h. The results revealed that the levels of cell adhesion of PC cell lines (22Rv1 or PC-3) were significantly decreased (Figure 3(A,B)). Furthermore, the wound healing assay also demonstrated an inhibitory effect of SDS on the migration of PC cell lines *in vitro* (Figure 3(C–F)).

**Figure 4.**
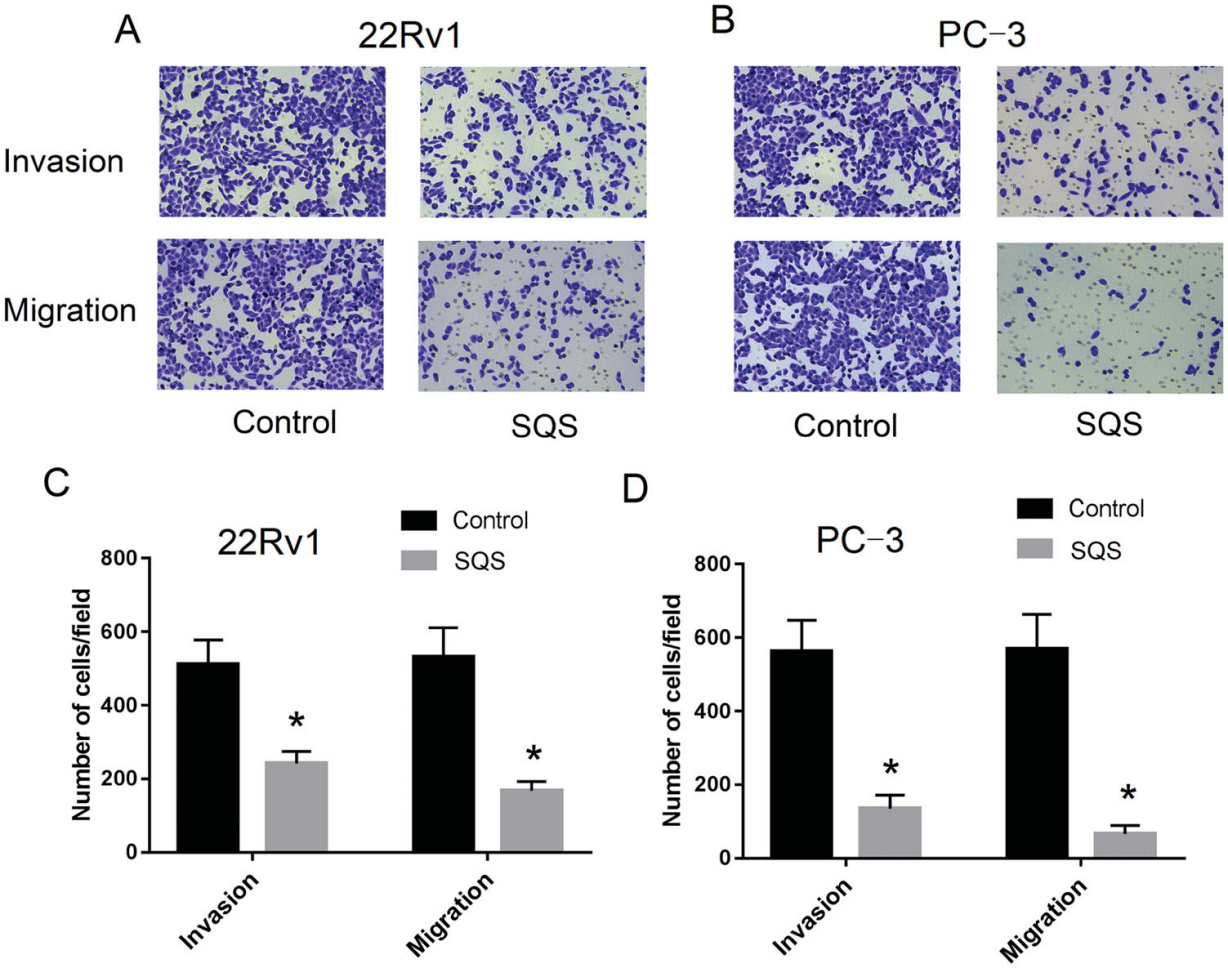
SQS inhibited the invasive ability of prostate cancer cell lines. Invasive ability of (A) 22Rv1 and (B) PC-3 cells after treatment with different concentrations of SQS. Number of cells/field of view of the chamber of (C) 22Rv1 and (D) PC-3 cells after treatment with different concentrations of SQS. Each experiment was performed in triplicate, and the data are presented as the mean ± SD. *p < 0.05 vs. control. SQS, sasanquasaponin.

Transwell invasion and migration assays were performed to evaluate the invasive ability of 22Rv1 or PC-3 cells. Upon stimulation with SQS, the cell number of stained crystal violet was significantly smaller, which indicated that the invasive and migration ability of the PC cell lines was reduced (Figure 4(A–D)). Collectively, these data indicated that SQS could inhibit the adhesion, migration, and invasion of PC cell lines *in vitro*.

### SQS inhibited the EMT of PC cell lines

**Figure 5.**
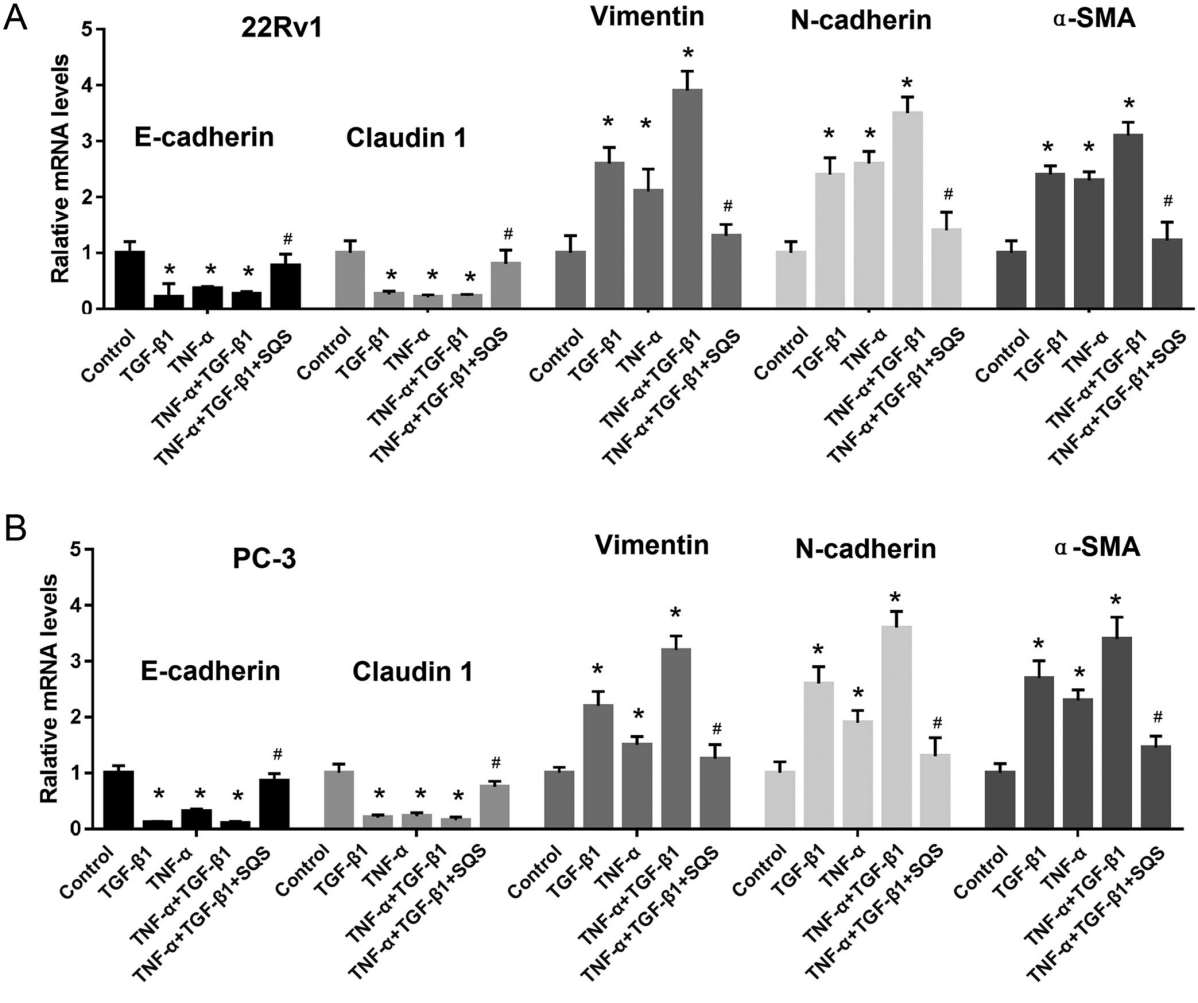
SQS reduced the mRNA expression levels of epithelial to mesenchymal transition-related genes in prostate cancer cell lines. mRNA levels of E-cadherin, claudin 1, vimentin, N-cadherin and α-SMA in (A) 22Rv1 and (B) PC-3 cells after treatment with SQS, TGF-β1, and/or TNF-α. GAPDH was used for normalization. Each experiment was performed three times, and the data are presented as the mean ± SD. *p < 0.05 vs. control; ^#^*p* < 0.05 vs. TNF-α + TGF-β1 group. SQS, sasanquasaponin; α-SMA, α-smooth muscle actin.

**Figure 6.**
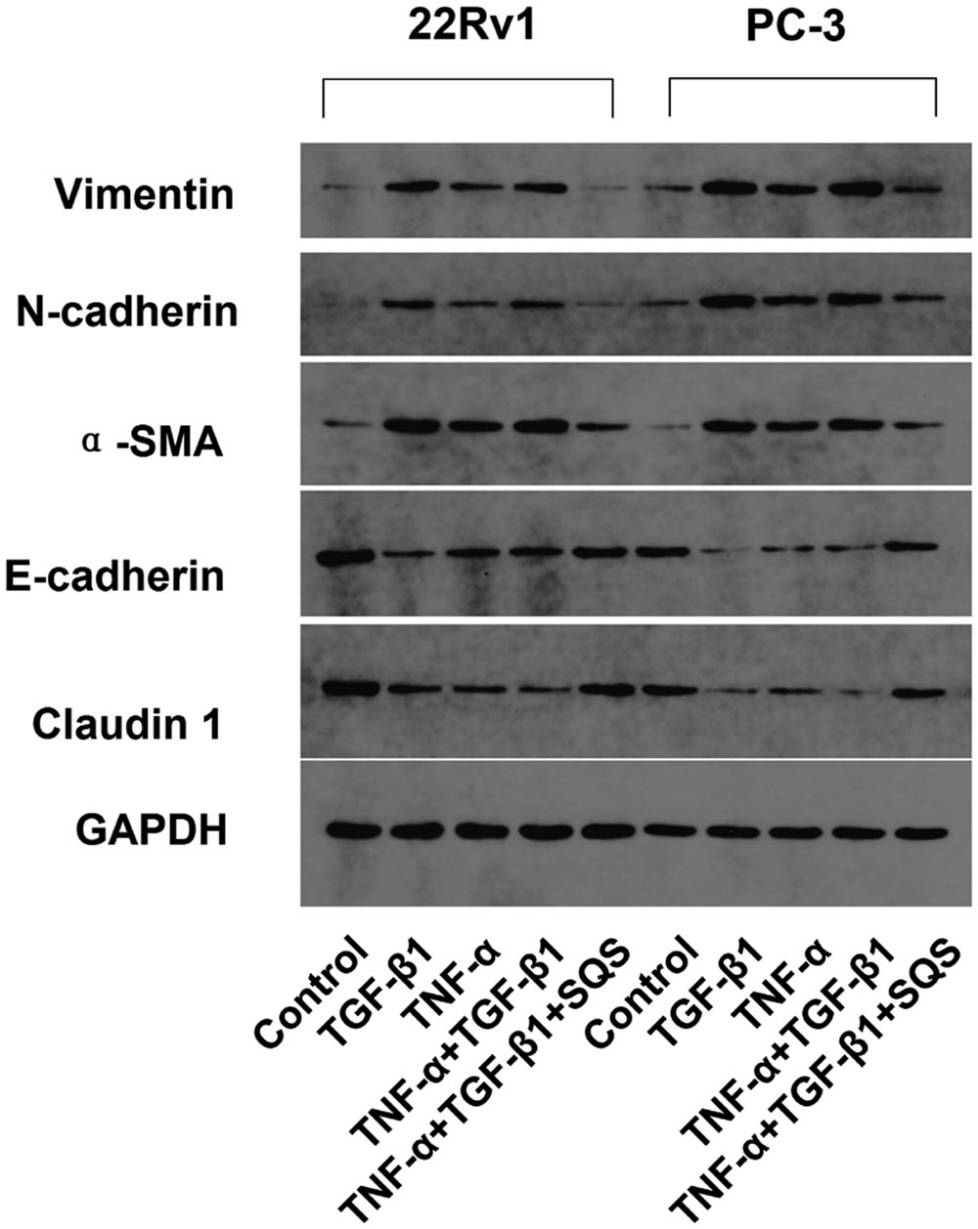
SQS inhibited the protein levels of epithelial to mesenchymal transition-related genes in prostate cancer cell lines. Protein levels of E-cadherin, claudin 1, vimentin, N-cadherin and α-smooth muscle actin in 22Rv1 or PC-3 cells after treatment with SQS, TGF-β1, and/or TNF-α. GAPDH was used for normalization. SQS, sasanquasaponin.

EMT has been reported as an essential mechanism associated with the invasion and metastasis of tumours (Lamouille et al. 2014). Thus, the function of SQS in the EMT of PC was examined. First, EMT was induced in 22Rv1 or PC-3 cells using TGF-β1 or TNF-α. Next, the impact of SQS on the EMT of PC cell lines was investigated. The results demonstrated that the mRNA and protein levels of EMT-related markers showed a significant change. After treatment with SQS, the expression of E-cadherin and claudin 1 was increased, while the expression of vimentin, N-cadherin, and α-smooth muscle actin was decreased in 22Rv1 or PC-3 cells (Figures 5 and 6). Thus, the present experiments suggested that SQS could inhibit the EMT of PC cell lines.

### SQS inhibited the migration and invasion of 22Rv1 cells induced by TGF-β1 or TNF-α

**Figure 7.**
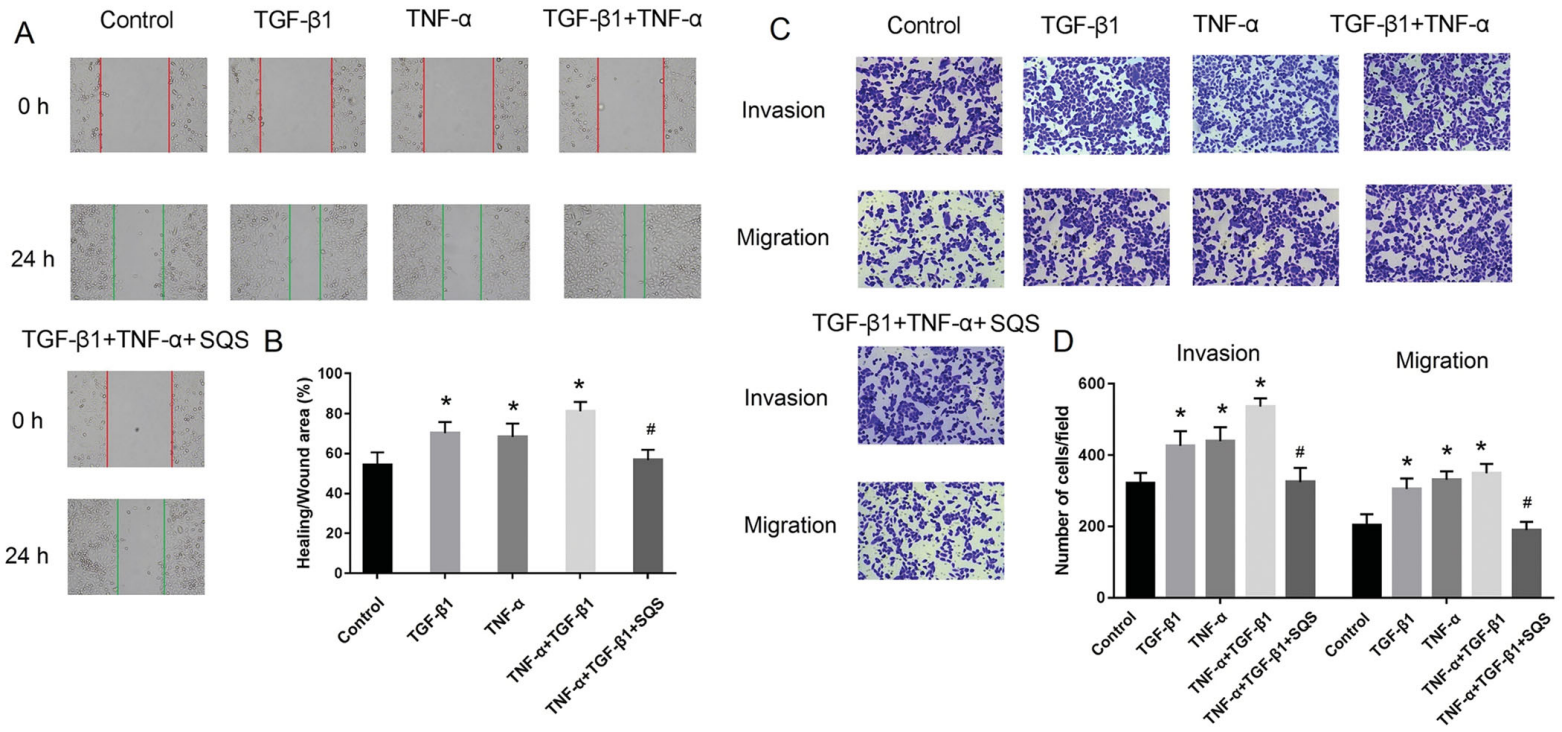
SQS inhibited the migration and invasion of 22Rv1 cells induced by TGF-β1 or TNF-α. (A) Migratory ability of 22Rv1 cells after treatment with SQS, TGF-β1, and/or TNF-α. (B) Ratio of healing to wounded areas in 22Rv1 cells after treatment with SQS, TGF-β1, and/or TNF-α. (C) Invasive ability of 22Rv1 cells after treatment with SQS, TGF-β1, and/or TNF-α. (D) Number of cells/field of view of the chamber of 22Rv1 cells after treatment with SQS, TGF-β1, and/or TNF-α. Each experiment was performed three times, and the data are presented as the mean ± SD. ^#^*p* < 0.05 vs. TNF-α + TGF-β1 group. SQS, sasanquasaponin.

TGF-β1 and TNF-α were used to induce EMT in PC cell lines, and whether the migration and invasion of 22Rv1 cells induced by TGF-β1 or TNF-α could be inhibited by SQS was next evaluated. The results of invasion and wound healing assays demonstrated that TGF-β1, TNF-α, or TNF-α + TGF-β1 could promote the migratory and invasive abilities of 22Rv1 cells. However, after treated with SQS for 24 h, the positive influence of TGF-β1 and TNF-α on the migration and invasion of 22Rv1 cells could be neutralized (Figure 7(A–D)), which indicated that SQS could inhibit the migration and invasion of 22Rv1 cells induced by TGF-β1 or TNF-α.

### SQS inhibited the EMT of 22Rv1 cells by regulating the PI3K/Akt/mTOR and Smad pathways

**Figure 8.**
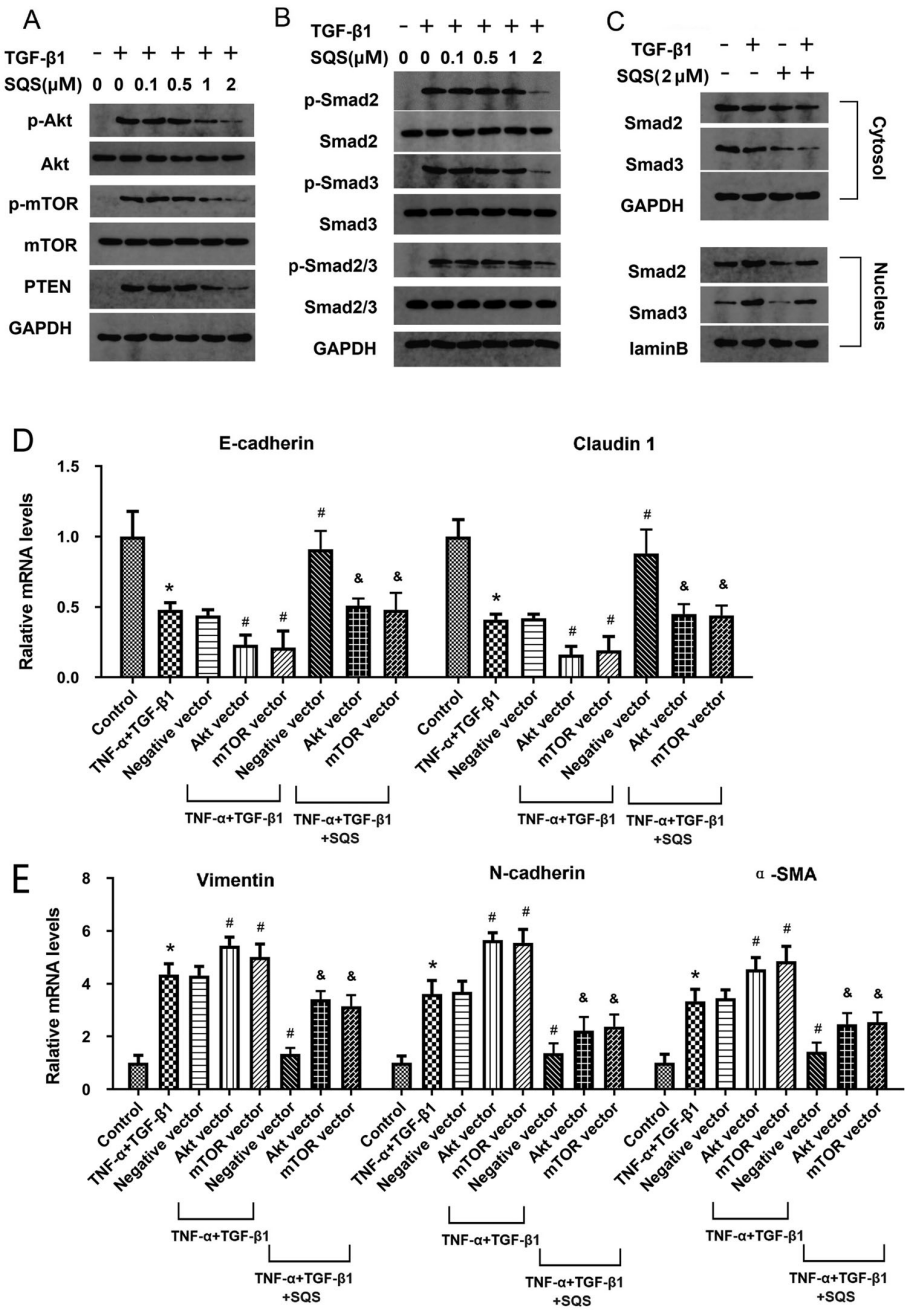
SQS inhibited the epithelial to mesenchymal transition of 22Rv1 cells by regulating the PI3K/Akt/mTOR and Smad pathways. (A) Activation of the Akt-mTOR axis in 22Rv1 cells after treatment with SQS and TGF-β1. GAPDH was used for normalization. (B) Activation of the Smad2/3 signalling pathway in 22Rv1 cells after treatment with SQS and TGF-β1. GAPDH was used for normalization. (C) Activation of the Smad2/3 signalling pathway in the nucleus or cytosol of 22Rv1 cells after treatment with SQS and TGF-β1. GAPDH was used for normalization. (D) mRNA levels of E-cadherin and claudin 1 in Akt or mTOR-overexpressing 22Rv1 cells after treatment with SQS, TGF-β1, and/or TNF-α. (E) mRNA levels of vimentin, N-cadherin and α-smooth muscle actin in Akt or mTOR-overexpressing 22Rv1 cells after treatment with SQS, TGF-β1, and/or TNF-α. GAPDH was used for normalization. *p < 0.05 vs. control; ^#^*p* < 0.05 vs. TNF-α + TGF-β1 + Negative vector group; ^&^*p* < 0.05 vs. TNF-α + TGF-β1 + SQS + Negative control group. SQS, sasanquasaponin.

Considering that PI3K/Akt and Smad positively regulate the process of EMT in several tumours (Martini et al. 2014; Chen et al. 2016; Tang et al. 2017; Su et al. 2020), the present study further explored whether these signalling pathways also play a function in the EMT of PC. As shown in Figure 8(A,B), the PI3K/Akt/mTOR and Smad2/3 signalling pathways were activated in the process of EMT in 22Rv1 cells induced by TGF-β1, and SQS could significantly reduce the activation of the PI3K/Akt/mTOR and Smad2/3 pathways. The level of activation of these two signalling pathways in the nucleus and cytosol of 22Rv1 cells was investigated, and it was found that SQS could exert an inhibitory impact on EMT in both the nucleus and cytosol of 22Rv1 cells (Figure 8(C)). Furthermore, as shown in Figure8(D,E), Akt and mTOR-overexpressing 22Rv1 cells were established by transfection with the corresponding plasmids, and it was found that the mRNA of EMT-related genes was changed in 22Rv1 cells after been treated with TNF-α + TGF-β1. After being treated with negative vector, the levels of mRNA of EMT-related genes were not significantly changed, but after cells were transfected with Akt and mTOR vectors, the levels of mRNA of EMT-related genes were significantly changed compared with Negative vector (E-cadherin and Claudin-1 were decreased; Vimentin, N-cadherin, and α-SMA were increased). When cells were treated with TNF-α + TGF-β1 + SQS, the mRNA levels of E-cadherin and Claudin-1 were significantly increased, while the mRNA levels of Vimentin, N-cadherin, and α-SMA were significantly decreased compared with TNF-α + TGF-β1 groups. Compared with TNF-α + TGF-β1 + SQS group, the TNF-α + TGF-β1 + SQS + Akt vector and TNF-α + TGF-β1 + SQS + mTOR vector treatments both significantly changed the mRNA levels of EMT-related genes, indicating that Akt and mTOR participate in the regulation role of SQS on EMT. In summary, the present data indicated that SQS could inhibit the EMT of 22Rv1 cells by regulating the PI3K/Akt/mTOR and Smad pathways.

### SQS inhibited the growth of xenograft tumours in vivo

**Figure 9.**
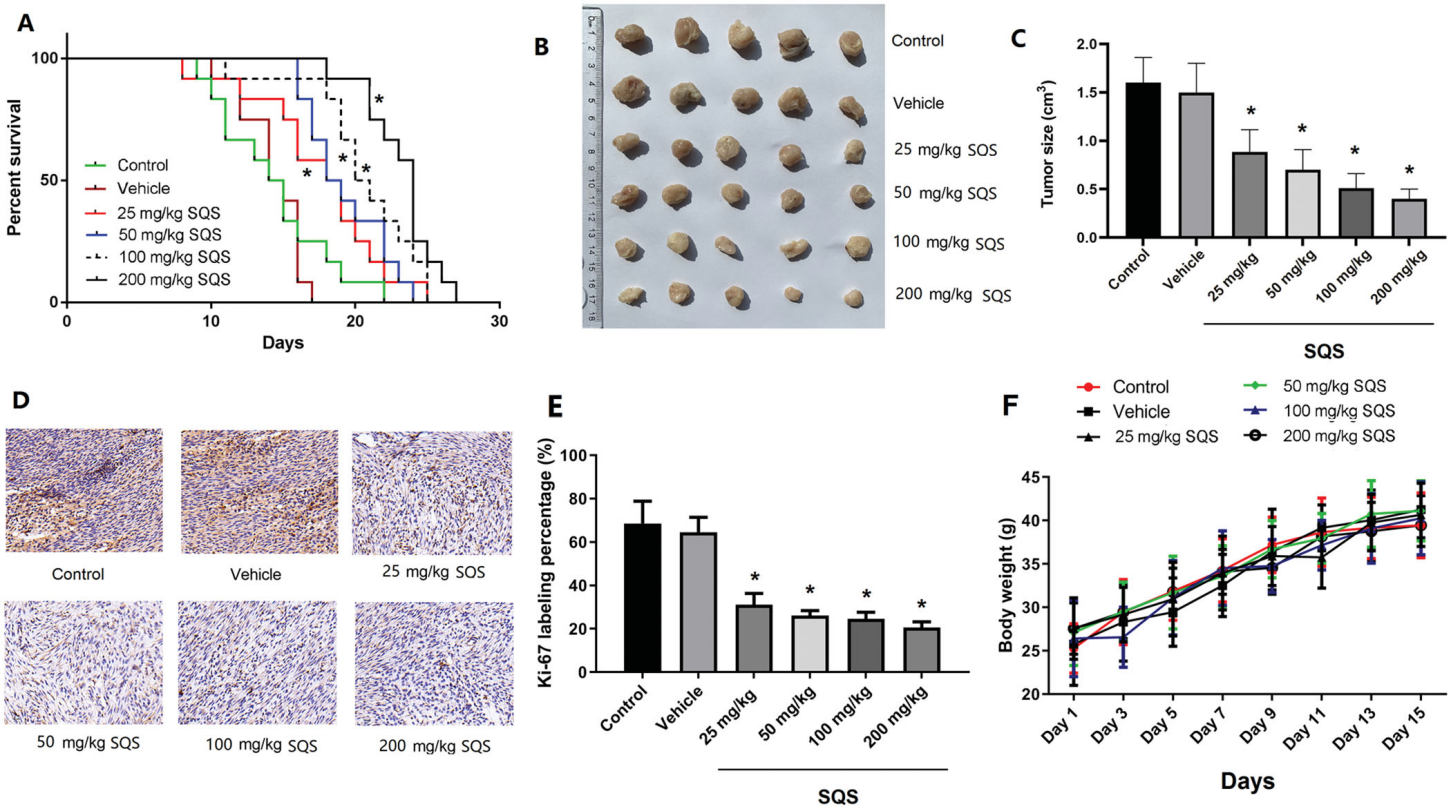
SQS inhibited the growth of xenograft tumours *in vivo*. (A) Percentage of survival of nude mice with xenograft tumours after intragastric treatment with SQS. (B) Images of xenograft tumours after intragastric treatment with SQS. (C) The size of xenograft tumours after treatment with intragastric administration of SQS. (D) Representative images of Ki-67 staining in xenograft tumours. (E) Ki-67 labelling percentage in groups. (F) Weight changes of mice. Each experiment was performed three times, and the data are presented as the mean ± SD. *p < 0.05 vs. control. SQS, sasanquasaponin.

Animal experiments were conducted to verify the inhibitory effects of SQS on PC therapy by using tumour xenograft studies. It was found that intragastric administration of SQS in nude mice exerted a positive impact on survival rate (Figure 9(A), *p* < 0.05). Furthermore, intragastric administration of SQS could also significantly prevent the growth of xenograft tumours, as indicated by the tumour size compared with that of tumours derived from control nude mice (Figure 9(B,C), *p* < 0.05). The Ki-67 staining was performed to evaluate the proliferation ability of tumour. The representative images are shown in Figure 9(D). The quantitative results are shown in Figure 9(E). The Ki-67 labelling percentage in the SQS groups were significantly decreased compared with Control (*p* < 0.05). The weight changes are shown in Figure 9(F). There was no significant difference between groups, indicating that the treatment of SQS did not cause toxicity in mice. The results of these animal experiments suggested that SQS could act as an effective antitumour agent *in vivo*.

## Discussion

Recently, biomedical research on PC has garnered increasing attention due to the rapid increase in the incidence and mortality of patients with PC, which was caused by an increase in the ageing of the population (Denmeade and Isaacs 2002). During to the early stages of PC, numerous patients miss the most appropriate time for diagnosis and treatment (Mohan and Schellhammer 2011). Regarding its pathology and clinical symptoms, PC is heterogeneous, varying from an indolent disease, to an aggressive and metastatic disease. Therefore, it is necessary to develop novel biomarkers for early diagnosis and therapy. For the first time, the present study revealed that SQS inhibited the proliferation, adhesion, migration, and invasion of 22Rv1 and PC-3 cells. SQS also inhibited the EMT of 22Rv1 and PC-3 cells induced by TGF-β1 or TNF-α. The PI3K/Akt/mTOR and Smad2/3 signalling pathways were activated in the process of EMT in 22Rv1 cells induced by TGF-β1, and SQS could significantly inhibit these pathways. Finally, SQS inhibited the growth of xenograft tumours *in vivo*. In total, these results indicated that SQS inhibited the growth and EMT in PC via PI3K/Akt/mTOR and Smad pathways.

Previous studies have revealed that traditional Chinese medicines show a positive effect on the treatment of various cancer types, including PC (Liu et al. 2016). SQS is a biologically active ingredient, which is obtained from the Chinese medicinal herb *C. oleifera* (Ye et al. 2014). Numerous studies have reported that SQS has a significant cardioprotective effect (Lai et al. 2004; Chen et al. 2007; Qiu et al. 2016). SQS was reported to have a good effect in various cancer types. For example, Liang et al. (2019) reported that SQS could induce autophagy and promote apoptosis to inhibit human melanoma via the Akt/mTOR/p70S6K pathway. Chen et al. (2013) also reported that SQS may contribute to the cell cycle arrest and apoptosis of human breast cancer cells *in vitro*. Furthermore, SQS treatment may inhibit the proliferation of liver cancer by activating Bcl-2, Bax, and caspase-3 (Zeng et al. 2015). However, the molecular basis for the SQS-based treatment of PC remains to be fully elucidated. The present study revealed that SQS may act as an efficient inhibitory agent for the treatment of PC. SQS could inhibit the viability, adhesion, migration, and invasion of PC cell lines *in vitro*. Additionally, SQS may inhibit the growth of xenograft tumours *in vivo*. These results preliminarily proved that SQS may act as an antitumour agent against PC.

Due to the difficulty of early diagnosis and the characteristics of heterogeneity in PC, cancer metastasis has become a marked obstacle in the clinical treatment of PC (Chang et al. 2014). Adhesion, migration, and invasion play important roles in the progression of cancer cell metastasis (Pavese et al. 2010). Therefore, restricting the capability of these metastasis-related characteristics could effectively control cancer cell metastasis. The present study revealed that SQS could reduce 22Rv1 and PC-3 cell adhesion, migration, and invasion *in vitro*. These results indicated that SQS may also inhibit the tumour metastasis of PC. Our next goal was to explore the underlying mechanism of the anti-metastasis effect of SQS. EMT was demonstrated to be an essential mechanism associated with the invasion and metastasis of tumours, which may cause the disruption of matrix junctions and apical–basal polarity in tumour epithelial cells (Lamouille et al. 2014; Odero-Marah et al. 2018). The present study found that SQS significantly inhibited the EMT of PC cell lines (22Rv1 or PC-3) induced by TGF-β1 or TNF-α. In summary, this evidence suggested that SQS could suppress the tumour metastasis of PC cells *in vitro* by inhibiting the EMT process. SQS could regulate the expression of EMT-related proteins, thus inhibit the metastasis ability of PC, but the exact mechanism needs further investigation.

PI3K/Akt, a crucial intracellular signal transduction pathway, is associated with the development and progression of various cancer types (Chen J 2010; Zhang et al. 2016). In numerous cellular biological processes, particularly in cell proliferation and metastasis, the PI3K/Akt signalling pathway is significantly activated (Ma et al. 2013). For instance, Shukla et al. (2007) reported that the PI3K/Akt signalling pathway could regulate the invasion of PC cells, which was also confirmed by the present study. The Smad signalling pathway is a novel signal transduction pathway that regulates multiple essential pathological processes in PC (Bello-DeOcampo and Tindall 2003). A recent study demonstrated that suppressing the Smad signalling pathway could inhibit PC pathogenesis after silencing paired box 3 (Zeng et al. 2020). The study by Zhao et al. (2018) revealed that the long non-coding RNA ANRIL was overexpressed in PC and promoted the proliferation and migration of PC, which was associated with the activation of the Smad signalling pathway. Based on these studies, the effect of SQS on the PI3K/Akt and Smad signal pathways was explored in the present study. When cells were treated with TNF-α + TGF-β1 + SQS, Akt vector and mTOR vector treatments both significantly changed the mRNA levels of EMT-related genes, indicating that Akt and mTOR participate in the regulation role of SQS on EMT. The current results suggested that the PI3K/Akt/mTOR and Smad signalling pathways may regulate the inhibitory impact of SQS-induced EMT in 22Rv1 cells. This is consistent with previously mentioned studies, which indicated that the proliferation and migration of PC was associated with the activation of the PI3K/Akt/mTOR and Smad signalling pathways. Thus, these emerging results indicate the potential of the PI3K/Akt/mTOR and Smad signalling pathways as novel therapeutic targets in patients with PC.

In this research, we chose two cell lines, 22Rv1 and PC-3 cells for study. Though the results of these two cell lines were similar, it still showed some differentiations between these two cell lines. Nowadays, it still loss specific reports to clarify the differentiation between 22Rv1 and PC-3 cells. However, cell lines from same diseases always showed different characteristics. PC-3 is a cell line initiated from a bone metastasis of a grade IV prostatic adenocarcinoma from a 62-year-old, White, male. 22Rv1 is a human prostate carcinoma epithelial cell line derived from a xenograft that was serially propagated in mice. Different origin and tissue type may be the reason of the different results of these two cell lines.

The present study has various limitations: (i) The specific signalling pathways that regulate EMT need to be investigated in the future; (ii) additional experiments are necessary, including the use of signalling pathway inhibitors; and (iii) it is necessary to explore the precise concentration of SQS and duration of treatment in future clinical research.

## Conclusions

To the best of our knowledge, the present study is the first to investigate the inhibitory effects of SQS on the treatment of PC. The current results demonstrated that SQS could inhibit the viability, adhesion, migration, and invasion of PC cell lines. Notably, it was revealed that the PI3K/Akt/mTOR and Smad signalling pathways were responsible for inhibiting the EMT of PC cell lines induced by SQS. Furthermore, SQS also inhibited the growth of xenograft tumours *in vivo*. Thus, these findings may be beneficial for the further understanding of the molecular mechanism associated with SQS-induced antitumour effects and for elucidating the cellular effect and pharmacological profiles of such effects.

## Author contributions

W.L. performed the experiments. Y.M. wrote the manuscript. B.H. analysed the data. X.G. performed the statistical analysis. C.L. conducted the literature research. X.B. and W.P. conceived and designed the study and reviewed/edited the article. All authors had read and approved the article.

## Supplementary Material

Supplemental Material

## Data Availability

The datasets used and/or analysed for this study are available from the corresponding author upon reasonable request.
