# Supplementary figures and images for: Sasanquasaponin inhibited epithelial to mesenchymal transition in prostate cancer by regulating the PI3K/Akt/mTOR and Smad pathways
Source: Pharm Biol. 2022 Oct 7;60(1):1865–75. doi: 10.1080/13880209.2022.2123931 (PMC9553173; doi:10.1080/13880209.2022.2123931)

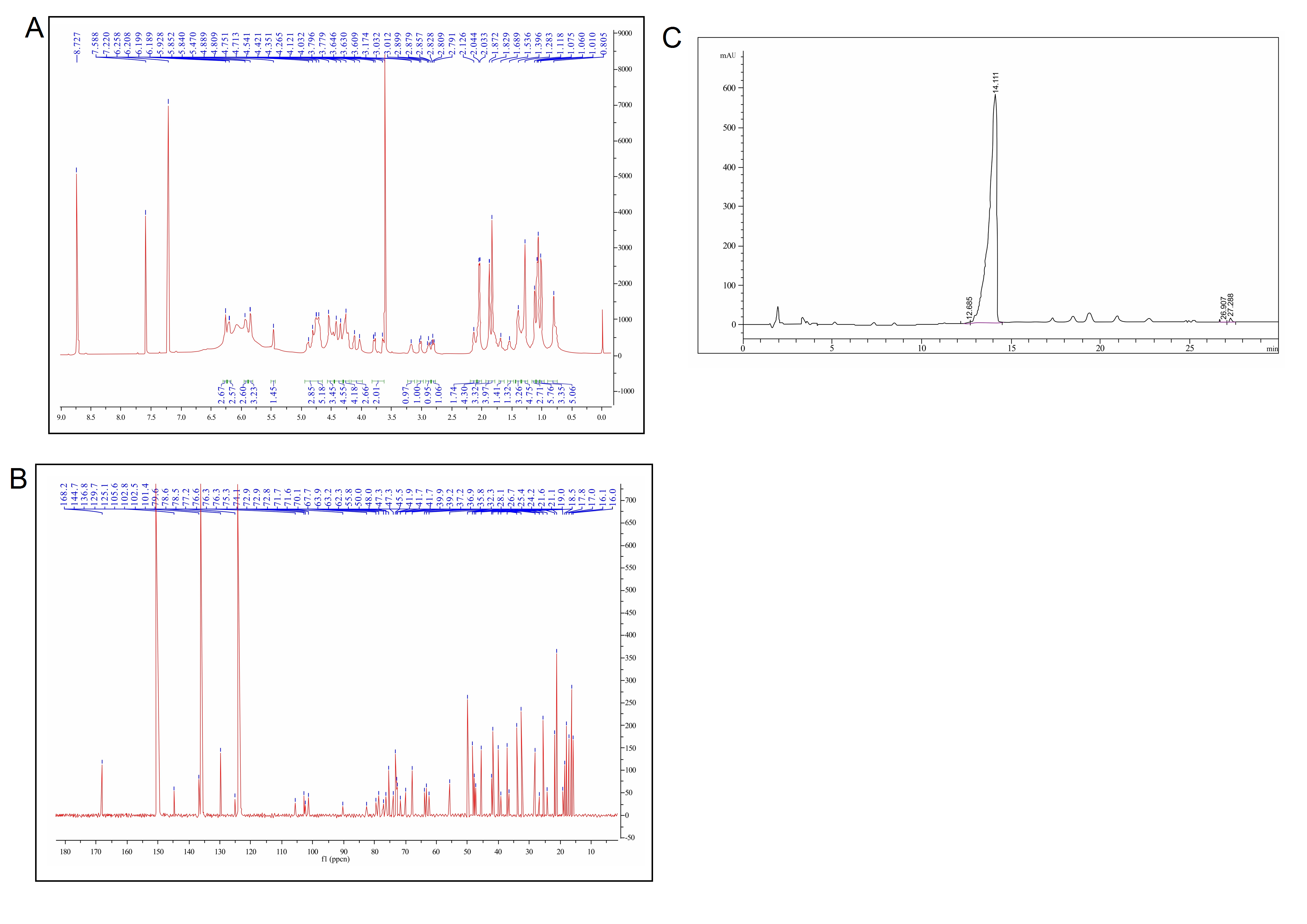

Supplement: Supplemental Material [file IPHB_A_2123931_SM5879.tif]
